# A technique to use CT images for *in vivo* detection and quantification of the spatial distribution of radiation‐induced esophagitis

**DOI:** 10.1120/jacmp.v14i3.4195

**Published:** 2013-05-06

**Authors:** Laurence E. Court, Susan L. Tucker, Daniel Gomez, Zhongxing Liao, Joy Zhang, Stephen Kry, Lei Dong, Mary K. Martel

**Affiliations:** ^1^ Department of Radiation Physics The University of Texas MD Anderson Cancer Center Houston TX; ^2^ Department of Bioinformatics and Computational Biology The University of Texas MD Anderson Cancer Center Houston TX; ^3^ Department of Radiation Oncology The University of Texas MD Anderson Cancer Center Houston TX USA

**Keywords:** esophagitis, radiation toxicity

## Abstract

The purpose of the study was to examine whether CT imaging can be used to quantify radiation‐induced injury to the esophagus. Weekly CT images for 14 patients receiving proton therapy for thoracic tumors were retrospectively reviewed. The images were registered with the original treatment planning CT image using deformable registration techniques, and the esophageal contours from the treatment plan were automatically mapped to the weekly images. The relative change in the size of the esophagus was calculated for each CT slice as the ratio of the cross‐sectional area of the esophagus (minus air) in the weekly CT image to the same area in the planning CT image. The maximum relative change in cross‐sectional area for each CT image was calculated and examined for correlation with the clinical toxicity score for all the patients. The average maximum relative expansion of the esophagus at the end of treatment was 1.41±0.26,1.68±0.36, and 2.10±0.18 for patients with grade 0, 2, and 3 esophagitis, respectively. An unpaired t‐test, with the level of significance corrected with a Bonferroni correction, showed that the difference between grade 3 and 0 was significant, but the differences between grade 0 and 2, and 2 and 3 were not. The timing of changes in esophageal expansion closely matched that of clinically noted changes in patient symptoms. Expansion of the esophagus on CT images has potential as an objective measure of toxicity. The ability to quantify objectively the spatial distribution of radiation‐induced injury will be a useful tool in understanding the impact of partial esophageal sparing on the probability of esophagitis.

PACS number: 87.55.dh

## I. INTRODUCTION

It is well known that there is a correlation between the dose to the esophagus during radiation therapy for lung cancer and acute esophagitis.^1^, ^2^ However, even patients whose radiation treatment plans are acceptable given our current dose‐volume constraint criteria can experience acute esophagitis. It has been reported that 15%–25% of patients receiving either concurrent chemoradiotherapy or hyperfractionation can experience severe acute esophagitis.^2^, ^3^, ^4^ This can necessitate surgical intervention, hospitalization, or breaks in radiation therapy, which can lower local tumor control. It can also be a dose‐limiting factor that prevents dose escalation to the tumor. Finally, acute esophagitis predicts long‐term esophageal sequelae that undermine patients’ ongoing quality of life.^(5)^ An excellent review of our current understanding of the dose‐response characteristics of the esophagus can be found in Werner‐Wasik et al.^(2)^

In some cases, it is impossible to completely avoid the esophagus because it lies within the target volume. However, our experience is that for many of our patients who experience moderate or severe esophagitis, the esophagus is actually outside or at the edge of the target volume, meaning there is potential for improving the esophageal dose distribution in these patients. Modern radiation therapy centers have the technical capability to sculpt dose distributions to avoid critical tissues (e.g., with IMRT, VMAT, or proton therapy), but the relationship between the spatial dose distribution and the spatial distribution of the esophagitis is complex and has not been elucidated. Until this relationship is well understood, it will not be possible to present a clear planning goal to the treatment planners and other clinical staff. Several groups have tried to evaluate this relationship^5^, ^6^ and understand the potential for esophagus‐sparing techniques, but detailed work is hindered because of our current inability to visualize the actual spatial distribution of radiation‐induced esophageal injury. Most studies, such as those reviewed in Werner‐Wasik et al.^(2)^ and others (e.g., Singh et al.^(7)^), seek to establish a link between radiation dose distribution (spatial dose distribution, DVH, or other descriptor) and reported symptoms, without being able to visualize the injury. The ability to visualize and quantify the actual tissue injury could help link the dose distribution to the injury and the injury to the reported symptoms. The primary goal of this initial work was to determine whether we can develop a technique to quantify the severity of esophagitis based on CT images. Such a tool would facilitate studies to investigate the issues described above.

## II. MATERIALS AND METHODS

### A. Source of CT images

The CT images used in this study were taken using two scanners: Philips Mx8000 IDT 16 (Philips Healthcare, Andover, MA), which acquired 4D CT using a helical scan approach, and GE LightSpeed RT16 (GE Healthcare, Waukesha, WI), which used an axial scan approach for creating of 4D CT images. Wherever possible, imaging parameters were kept identical (120 kVp, no contrast, 2.5 mm slices, 0.98 mm in‐plane voxels). Images were acquired as part of a 4D CT protocol investigating inter‐ and intrafraction motion, approved by the Institutional Review Board at the University of Texas MD Anderson Cancer Center, with patient consent (protocol number: 2003–0962).

### B. Treatment plans

The patients examined in this study were treated with proton therapy using 2–3 coplanar beams. Their treatment plans were created using the Eclipse treatment planning system (Varian Medical Systems, Palo Alto, CA). For each field, a block was designed to shape the field and a compensator designed to conform the distal edge of the dose distribution to the distal surface of the planning target volume (PTV). The PTV margin includes geometric uncertainties, but also accounts for other uncertainties (specifically uncertainties in the proton range). Dose distributions were calculated using a dose grid resolution of $2\times 2\times 2.5\;{mm}^{3}$, using the pencil beam convolution algorithm which is commissioned in Eclipse. For a detailed discussion of the proton planning process, readers are referred to Kang et al.^(8)^ and Chang et al.^(9)^

### C. Analysis of changes to the esophagus

**Figure 1:**
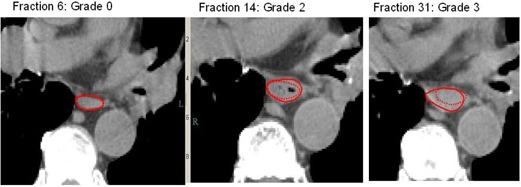
Weekly CT images for Patient A. The dotted contour shows the shape of the esophagus in the treatment plan. The solid contour shows the current shape of the esophagus, from deformable registration. Expansion of the esophagus can be seen from fraction 14, corresponding with the esophagitis grade in the patient record.

Deformable image registration techniques^(10)^ were used to register the exhale‐phase CT images (from 4D CT image sets) taken before treatment with images taken during treatment in the same exhale phase. The use of images from the same phase will minimize the impact of any inter‐ or intrafractional motion on the position or shape of the esophagus. The original esophageal contour was automatically mapped to the new CT image. An example of this is seen in Fig. 1, where the original contour (dotted line) is mapped to the new CT using deformable registration, giving the new esophagus contour (solid line). The esophagus’ change in size was calculated as the ratio of its cross‐sectional area in the weekly CT image to that in the pretreatment CT image, using thresholding techniques to remove air from the area calculation. This means that, for example, the air inside the esophagus for the patient in Fig. 1 does not result in an incorrect increase in cross‐sectional area for fraction 14. This approach was necessary because the esophagus often appears to be collapsed (i.e., no air), but this varies from day to day. We used cross‐sectional area of the esophagus as a surrogate for changes in wall thickness because the esophageal wall cannot be well visualized in noncontrast CT images. The calculations were repeated for each craniocaudal location (CT slice position) in the esophagus, giving a one‐dimensional output that showed the variation of esophageal cross‐sectional area with craniocaudal position. The results from adjacent slices were then averaged to help reduce apparent abrupt steps in cross‐sectional area between adjacent CT slices caused by varying undulations/folds in the esophagus. That is, the results for a single slice is presented as the average of the results for that slice, the slice above, and the slice below.

This technique was used to calculate the maximum relative change in cross‐sectional area of the esophagus for 14 patients who had been treated for lung cancer using proton therapy. This metric was then compared with the clinical toxicity score which was extracted from the patients’ medical records. The Common Terminology Criteria for Adverse Events (v.3) grading of esophagitis was used.(11) Under this system, grade 1 corresponds to asymptomatic pathologic, radiographic, or endoscopic findings only, so is not used here as we do not have that data. Any symptom, such as altered eating or swelling, is labeled as grade 2. More severe symptoms, such as severely altered eating/swelling or the need for IV fluids for more than 24 hours, are labeled as grade 3. The patients were preselected to give a range of esophagitis grades. Clinical esophagitis grades at the end of treatment were: grade 0: four patients; grade 2: seven patients; grade 3: three patients. All patients received concurrent chemotherapy. All patients had 200 cGy fractions to a median total dose of 7400 cGy (range: 6300–7400 cGy). None of the patients were on a course of steroids (other than inhaled steroids for COPD) during their radiation therapy. The change in the cross‐sectional area of the esophagus through the course of treatment was closely examined using weekly CT images each for all these patients.

## III. RESULTS

The average maximum expansion of the esophagus at the time of maximum esophagitis grade was 1.30±0.21,1.72±0.36, and 2.10±0.18 for patients with grade 0, 2, and 3 esophagitis, respectively. An unpaired t‐test, with the level of significance corrected with a Bonferroni correction to account for the fact that multiple hypotheses were examined (a=0.05/3 hypotheses=0.017), showed that the difference between grade 3 and grade 0 was statistically significant (p=0.005), but the differences between 0 and 2, and 2 and 3 were not (p=0.18, and 0.05, respectively).

**Figure 2:**
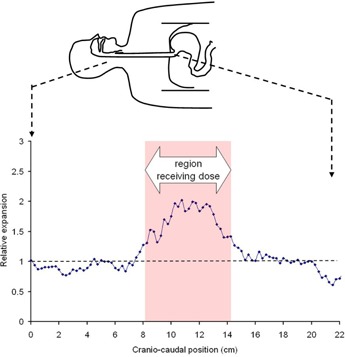
Relative expansion of the esophagus as a function of craniocaudal position for a patient with grade 3 esophagitis. The esophagus expands in the PTV region (shaded), but is unchanged outside this region.

The progression of esophageal expansion as the treatment progressed for one patient in shown in Fig. 1. Figure 2 shows the relative esophageal expansion as a function of craniocaudal position for one patient with grade 3 esophagitis. It can be seen that the length of the esophagus within the PTV (shaded region) expanded, but the esophagus outside this region was unchanged.

**Figure 3:**
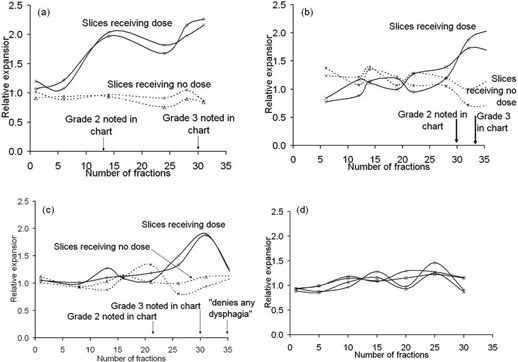
Variation of relative esophageal expansion with treated fraction for four patients: (a), (b), and (c) are for patients who experienced grade 3 esophagitis, (d) is for a patient who reported no symptoms of esophagitis. The solid lines are from two CT slices that received radiation dose. The dotted lines are for two slices that received no radiation dose. The esophagus is seen to expand around the time that the patient first experiences symptoms. The esophagus that receives no dose is unaffected.

Figures 3(a) to 3(c) show the progression in relative expansion of the esophagus for three patients who exhibited grade 3 esophagitis. The timing of changes in esophageal expansion for individual patients closely matched the clinically noted changes in toxicity. In each case, the increase in esophageal expansion occurred around the time that esophageal toxicity was first noted on the chart (grade 2). Note that the esophageal thickening for the third patient decreased in the final week. This followed their chemotherapy being put on hold. Their medical record noted that the patient denies any dysphagia, but also noted that they were receiving most nutrition via gastrostomy tube.

Figure 3(d) shows the variation in relative expansion as the treatment progressed for a patient without any symptoms of esophagitis. The average interfraction variation (single standard deviation) in relative esophageal expansion for individual slices when there was no change in patient symptoms was 0.14 (all patients). In each case, the deformed contours were carefully examined and no gross errors were found.

The length of the involved esophagus was also examined. The average continual length of esophagus for with the relative expansion was greater than 1.5 was 0.0±0.3 cm,0.9±1.5 cm, and 6.7±0.6 cm for patients with grade 0, 2, and 3 esophagitis, respectively. An unpaired t‐test showed that the difference between grade 3 and grade 0 or 1 was statistically significant (p<0.01 in both cases)

## IV. DISCUSSION

We have demonstrated that the analysis of CT images can be used to quantify esophagitis. All three patients who exhibited grade 3 esophagitis had associated relative esophageal expansions close to a factor of 2, and the involved length of esophagus was much larger than for lower grades. The differences between grade 2 and grade 0 were much less clear. We did find that the timing of the increase in relative expansion closely matched observations in the patients electronic record.

There are several sources of noise in our analysis. One of the largest of these is likely to be caused by uncertainties in assigning the esophagitis grades caused by interphysician and interpatient differences, and difficulties in retrospectively interpreting the patient record. Also, it is likely that the distinction between grades includes more factors than just the maximum relative expansion of the esophagus — for example, the length of time since onset of symptoms. There is also some potential bias in the results because of the way the patients were selected to have a relatively high percentage of grade 3 esophagitis.

Uncertainties in the deformable registration will contribute to uncertainties in the relative expansion data. Based on visual examination, the deformable registration worked well for the majority of cases. The one region where the registration does not always work well is the inferior portion of the esophagus where CT contrast is not sufficient for the task. For the patients studied here, this region was away from the treated area. If a future study desired data from that region, it may be necessary to use contrast‐enhanced CT images. For the rest of the esophagus, however, visual inspection indicated that the accuracy of the registration is better than 1 mm (1 pixel), although this was not quantified. This experience is comparable to the results previously reported for this algorithm for head‐and‐neck and prostate cases, where it was shown that 96% of deformed voxels were accurate within 2 mm.^(10)^ To put this into perspective, if we examine a case where the deformable registration incorrectly expands 25% of the outer edge of the esophagus by 1 mm, this would appear (for a 2 cm diameter esophagus) as an apparent esophageal expansion of 5%, which falls within the ranges of values found for each grade of esophagitis.

Finally, some of the noise in the data may be caused by the varying undulations/folds in the esophagus causing an apparent increase in cross‐sectional area. In the presented technique, the impact of abrupt changes is reduced by averaging the cross‐sectional area between slices, but an improvement might be to calculate the central path along the esophagus, and then evaluate the esophagus’ size in planes perpendicular to this path. This work may help improve the ability of the tool to distinguish between grade 0 and grade 2 esophagitis.

To our knowledge, the only study into the use of CT images to assess esophagitis was carried out by Berkovich et al.,^(12)^ although the authors did not stratify the data into esophagitis grades. They evaluated the CT findings in 29 patients who had undergone CT (for different purposes) within one month of a diagnosis of esophagitis. They found that the mean esophageal wall thickness was 4.7 mm (standard deviation (SD)=2.0 mm) for patients with esophagitis, compared with 2.9 mm (SD=0.8 mm) for control patients. Comparison of data is difficult because of differences in the analysis techniques. For example, we were unable to identify the esophageal wall in the available images and, therefore, used relative change in cross‐sectional area to describe the anatomical changes. A difference in the distribution of grades in the two groups also makes comparison difficult. The Berkivoch study did not find changes in the wall thickness for all patients with esophagitis. Similarly, there was a substantial overlap in the relative expansion for grade 0 and grade 2 patients in the study. The uncertainties in our analysis have been described above.

**Figure 4:**
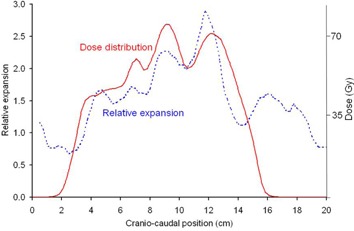
Spatial dose distribution and spatial distribution of esophageal expansion. The solid (red) line shows the dose distribution along the center of the esophagus as a function of craniocaudal position. The dotted line shows the relative expansion of the esophagus (compared with pretreatment CT images) as a function of craniocaudal position. Note that the shape of the dose distribution reflects the shape of the esophageal expansion.

**Figure 5:**
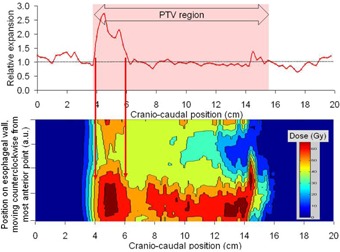
Relationship between the spatial distribution of esophageal expansion and the dose to the esophageal wall for one patient (with grade 2 esophagitis). The region of esophageal expansion is in the same physical location as a region where most of the esophageal wall receives a high dose (red arrows). Although more than 10 cm of the esophagus length received more than 50–60 Gy, there is no expansion in the esophagus for much of this length.

The use of these tools may overcome some of the issues experienced when researchers attempt to correlate dose parameters with reported toxicity grades. The evaluation of esophagitis with CT imaging, as reported here, could potentially overcome two pitfalls in assigning toxicity grades that were identified by the recent QUANTEC review:^(2)^ 1) Identifying the exact anatomic location of the esophagitis will help differentiate radiation‐induced esophagitis from other disorders with similar symptoms (infection, pre‐existing gastroesophageal reflux, or treatment‐related gastritis); and 2) being able to quantify objectively the toxicity grade will reduce interphysician differences in assigning grade 2 versus grade 3 endpoints. With CT imaging, the endpoints used for further study of esophagitis can be objective and may yield fewer false‐positive data points. Furthermore, these techniques may facilitate future studies into the relationship between the spatial dose distribution and the extent and magnitude of radiation‐induced esophagitis. To illustrate this, the dose distribution in the original plan was extracted from the treatment planning system, and the dose to the esophagus (based on the original contours) was then calculated in two ways: (i) the dose to the center of the esophagus for each CT slice, and (ii) the dose distribution to the esophageal wall. The dose to the esophageal wall was calculated by superimposing the esophagus’ contours onto the dose grid extracted from the treatment planning system. Each CT slice was treated separately, and the dose at 80 evenly spaced points around the contour was interpolated from the dose grid, starting with the most anterior point of the contour and moving counterclockwise. For both techniques, the dose was compared with the relative esophageal thickening on a slice‐by‐slice basis. Figure 4 shows the dose to the center of the esophagus and the spatial distribution of esophageal expansion for a patient with grade 3 esophagitis. Note that the shape of the dose distribution reflects the shape of the esophageal expansion. Figure 5 shows the relationship between the spatial distribution of the esophageal expansion and the dose to the esophageal wall for one patient (with grade 2 esophagitis). The region of esophageal expansion is in the same physical location as the region where most of the esophageal wall receives a high dose (red arrows). Although more than 10 cm of the esophagus length received more than 50–60 Gy, there is no expansion in the esophagus for much of this length. Of course, these are just single‐patient examples of potential uses of this technique. A full evaluation of the relationship between the spatial dose distribution and the tissue injury as characterized using esophageal thickening seen in CT images would require a much larger patient group than studied here, ideally in a prospective trial.

## V. CONCLUSIONS

Expansion of the esophagus on CT images has potential as an objective measure of toxicity. The ability to quantify objectively the spatial distribution of radiation‐induced injury may be a useful tool in understanding the impact of partial esophageal sparing on the probability of esophagitis which, in turn, may help create radiation therapy treatment plans with a reduced probability of esophagitis.

## ACKNOWLEDGMENTS

We would like to thanks Dr Sam Beddar and Dr Tina Briere for their support and advice throughout this work, and Kathryn Carnes for editorial support.
